# The Effect of Combining Mindfulness-Based Cognitive ‎Therapy with Pharmacotherapy on ‎Depression and Emotion ‎Regulation of Patients with Dysthymia: A Clinical Study

**Published:** 2016-07

**Authors:** Sajedeh Hamidian, Abdollah Omidi‎, Seyyed Masoud Mousavinasab, Ghasem Naziri

**Affiliations:** 1 Master of Clinical Psychology, Department of Psychiatry, Shiraz University of Medical ‎Sciences, Shiraz, Iran.; 2 Associate Professor, Department of Clinical Psychology, Kashan University of Medical Sciences, Kashan, Iran.; 3 Professor, Department of Psychiatry, Shiraz University of Medical ‎Sciences, Shiraz, Iran.; 4 Assistant Professor, Department of Psychology, Shiraz Branch, Islamic Azad University, Shiraz, Iran.

**Keywords:** *Dysthymia*, *Emotion Regulation*, *Mindfulness-Based Cognitive Therapy*

## Abstract

**Objective: **Mindfulness skills are assumed to be related with emotions. Deficits in emotion ‎regulation could lead to development and persistence of mood disorders. Dysthymia and double ‎depression are two chronic types of depression. This chronicity can be attributed to the one’s ‎inability to regulate his/ her mood. In this study, we aimed to evaluate the effect of mindfulness-‎based cognitive therapy (MBCT), which is one of the proposed methods for emotion regulation, ‎on depression and the ability of emotion regulation of patients with dysthymia.‎

**Method: **This clinical trial was conducted on 50 dysthymic and double ‎depressed patients. They were selected through convenience sampling and assigned into ‎intervention and control groups. The control group received only medication, while the MBCT ‎group participated in an eight- session program once a week with each session lasting for two to ‎two and half hours in addition to receiving medication. All the participants filled out Beck ‎Depression Inventory II and Difficulties in Emotion Regulation Scale before and after the ‎program. Data were analyzed using the SPSS statistical software (Version 16) and univariate ‎covariance statistical method.‎

**Results: **While there were no statistically significant differences between the two groups with ‎respect to the demographic characteristics, we observed a statistically significant improvement in ‎the defined variables in post-test of the MBCT group compared to the case group.‎

**Conclusion: **The results of this study revealed that combining MBCT and pharmacotherapy ‎could cause significant improvement in depression symptoms and increase the patient’s ability to ‎regulate emotion compared to pharmacotherapy alone‎.

## Introduction

The concept of depression has been recognized among the physicians. Avicenna, in his book ‎Canon of Medicine, referred to depression as “melancholy” and defined it as a disorder in which ‎the thought deviates from the natural path leading to destruction and fear. He believes that this ‎disorder is caused by ill-tempered dry and cold humor. In his opinion, dry and cold humor is ‎opposed to and hurts the spirit (1).‎

Dysthymia can be initially differentiated from major depression by its chronic nature and less ‎severe symptoms. Among the signs considered for major depression in DSM-IV, only ‎psychomotor disorder and suicide thoughts cannot be observed in dysthymia. This is somewhat ‎in line with the studies conducted by Beck et al. (1987) who reported suicide thoughts and loss ‎of appetite not to be the features of dysthymia (2).‎

A high rate of comorbid disorders are observed in this disorder, which is usually accompanied by ‎other psychological disorders such as anxiety disorders, drug abuse, and alcohol abuse. In ‎addition, more than 75% of individuals with dysthymia experience exacerbations in their ‎symptoms in the form of major depressive disorder (3-7), which is defined as “double ‎depression” (7, 8). Following the signs of dysthymia predicts strong risk factors for major ‎depression for both children and adults (7).‎

Emotion regulation or control has been a central concept for many Asian and European ‎philosophers. For instance, Spinoza, a 17th century philosopher, was highly interested in emotions; ‎he differentiated the negative emotions and positive ones and emphasized the regulation of the ‎passions. Studies by Block and Block (1980) on ego resilience directly affected the present ‎works on emotion regulation. Block emphasized the inefficient nature of over-controlling and ‎importance of flexible, optimum control for more compatibility (9).

Mood disorders are accompanied by disorders in perception and processing of the emotional ‎information and storing it in the memory. These disturbances may play a role in providing the ‎grounds for problems in interpersonal behaviors as well as the commencement and continuation ‎of mood disorders (10).‎

Some concepts in emotion regulation have emphasized controlling the emotional experiences, ‎emotional expression, and particularly severe control of negative emotions and reduction of ‎emotional arousal. On the other hand, some others have emphasized the functional nature of the ‎emotions in conceptualization of emotion regulation, suggesting that emotion regulation is not ‎similar to emotion control and does not mean immediate reduction in negative emotions. Hayes ‎et al. (1996) stated that making attempts to avoid internal experiences such as unwanted thoughts ‎and emotions provides the grounds for a large number of psychological disorders. In addition, ‎trying to control or suppress the emotional experiences may increase emotion regulation ‎disorders. Thus, these recent conceptualizations of emotion regulation emphasize the importance ‎of acceptance and valuation of the individuals’ emotional responses (11).‎

This is what mindfulness approaches do by completely focusing on the present experiences ‎through a moment-based approach. Mindfulness-based Cognitive Therapy (MBCT) combines ‎aspects of Beck’s cognitive therapy with meditations to train people to become more aware of ‎their bodily and mental experiences such as thoughts, feelings and external environments without ‎judging them. MBCT enables people to view their thoughts and emotions as transient phenomena ‎rather than facts (12). On the contrary to Beck’s cognitive therapy, MBCT does not aim to ‎correct the cognitive errors; it removes the thoughts from consciousness and help the individuals ‎to abandon the troublesome situations when immediate change is not possible (13, 14). Baer (2006) believes that being in a mindful status facilitates the exposure and prevention of responses ‎in emotional as well as psychological conditions. Similarly, Linehan (1993 a) states that ‎mindfulness practice may be beneficial for the individuals who fear from facing their emotions ‎‎ (15).‎

The MBCT method was planned initially to prevent relapse in depression, and a large number of ‎studies have shown its impact on the acute phase of depression and treatment-resistant ‎depressions (16-21).

Our target sample in this study was a group with chronic depression. Given the burden that this ‎type of depression causes the individual and society, choosing the most effective treatment plan ‎to deal with dysthymia and double depression is important and yet controversial. While some ‎studies have shown the superiority of SSRIs to psychotherapy alone in dysthymic patients (22), some others have emphasized the rapid and robust effect of medication and its little role in ‎reducing risks once it is discontinued (23). Imel et al. (2008) found that psychotherapy has ‎significant benefits at follow-up and prophylactic effects not retain by medication (24) ‎However, the majority of researches have suggested combined treatment as having the most ‎improvement rate compared to each of these methods alone (22, 25 and 26). In the case of MBCT, ‎Kuyken (2008) suggests that adding MBCT to usual treatment with antidepressants significantly ‎reduced residual symptoms and relapse/ recurrence rates of depression over 15-month follow-ups (27).‎

According to the mentioned studies, it seems that adding psychotherapy to pharmacotherapy in ‎the treatment of patients with dysthymia could have an additive effect. In this study, we aimed ‎to evaluate MBCT method to achieve the positive results in a group of patients with chronic ‎depression. ‎

## Materials and Method

***Participants***

Using convenience sampling, we selected 75 patients out of all patients who referred to two of ‎the psychiatric clinics affiliated to Shiraz University of Medical Sciences. These individuals had ‎been regularly referred to the collaborator psychiatrists during their course of disorder and were ‎diagnosed to have dysthymia or double depression disorder. A master of clinical psychology ‎interviewed the participants using SCID-I to ensure they met the diagnostic and inclusion criteria ‎for this study. According to the study criteria, 50 individuals were entered into the study and ‎randomly assigned to control and case groups. The case group received MBCT in addition to ‎medication, while the control group received medication only. All the study participants had ‎been receiving antidepressants of any type depending on the psychiatrist’s clinical judgment for ‎at least six months before the study. They participated in the study without changing the type ‎and dosage of medication they have been using treatment as usual (TAU). All the participants signed the informed ‎consent form before entering the research project.‎

The inclusion criteria of the study were as follows: Suffering from depression for at least two ‎years based on the DSM-IV-TR diagnostic criteria of dysthymia or double depression; being ‎older than 18 yrs.; having at least a high school diploma; not receiving any other psychological ‎treatment elsewhere such as individual psychotherapy that could coincide with the study; not ‎being diagnosed with severe mental disorders such as psychosis, mania, or a full criteria ‎personality disorders in the clinical interview; lack of drug or alcohol abuse while taking part in ‎the study; and not suffering from depression resulting from a simultaneous physical problem. ‎Moreover, the participants had to attend to at least five to eight treatment sessions.‎

***Procedure*** ‎

This study was conducted with a two-group quasi-experimental or static group comparison ‎design to evaluate the effect of MBCT program on emotion regulation and treatment of patients ‎with dysthymia who received medication.‎

***Measures***

Beck Depression Inventory-2 (BDI-II): This 21-item self-report questionnaire is a revised version ‎of BDI questionnaire that was developed to measure depression, and it is more consistent with ‎DSM-IV-TR compared to the first edition. BDI-II is used to assess the severity of depression in ‎people older than 13 years. Furthermore, Mohammadkhani and Dobson (2007) showed BDI-II as ‎a valid and reliable instrument for the Iranian population. The reliability coefficient for all the 21 ‎items was 0.913. The correlation of each item with the questionnaires indicated that item 15 (lack ‎of energy) with a coefficient of 0.618 had the highest possible recognition and item 19 (difficulty ‎concentrating) with a coefficient of 0.454 had the least ability to diagnose. To determine the ‎construct validity of the questionnaire, the correlation between the BDI-II and Depression Scale ‎of Brief Symptom Inventory was calculated to be 0.87 (28)‎‏.‏

Difficulties in Emotion Regulation Scale: Gratz developed this scale in 2004. It is a 36-item, ‎multidimensional, self-report questionnaire, which evaluates the individual’s emotion regulation ‎patterns and includes six subscales. This scale has been designed based on the experimental and ‎conceptual works that define emotion regulation as follows: Awareness and perception of the ‎emotions; acceptance of the emotions; the ability to control the impulsive behaviors and trying to ‎achieve goals while experiencing negative emotions; the ability to use flexible emotion ‎ regulation strategies to regulate the emotional responses for the individuals to reach their goals ‎and situational needs. Problem in each of these domains could indicate a disorder in emotion ‎regulation. ‎

This scale assesses six subscales including not accepting the emotional responses, problem in ‎performing the behaviors leading to one’s goal, impulse control problems, lack of emotional ‎awareness, limitation in finding emotion regulation strategies and lack of emotional clarity (29).‎

Sharifi et al. (2009) standardized this questionnaire in Iran. The reliability of the questionnaire ‎was calculated using bisection and Cronbach's alpha methods to be 0.86 and 0.8, respectively for ‎the whole questionnaire (P<0.01), indicating acceptable coefficients for the questionnaire. To ‎determine the concurrent validity of the questionnaire, its scores were correlated with the scores ‎of Zuckerman Sensation Seeking Scale, and a significant positive correlation was found between ‎them (n = 59, r = 0/26, p = 0.43). ‎

Structured Clinical Interview for Diagnosing Axis I Disorders in DSM-IV-TR (SCID-I): This ‎interview was designed by First, Spitzer, Gibbon, and Williams (2002), and it is used for ‎diagnosing dysthymia and refusing differential diagnoses. It has appropriate reliability and ‎validity for diagnosing psychological disorders. Sharifi et al. (2004) calculated psychometric ‎properties of the questionnaire for the Iranian population. Diagnostic agreement with clinical ‎interview by two psychiatrists according to Kappa index was above 0.6. Total Kappa index for ‎all current diagnosis was 0.52 and it was 0.55 for lifetime diagnosis (30).‎

***Treatment Process***

Segal et al. derived the program protocol from “MBCT for depression” (2002), which is ‎displayed in Table 1. It included eight weekly sessions with the duration of two to two and half ‎hours. Clinical interview was performed in a briefing session before the 8th session, and the ‎participants were evaluated in terms of inclusion and exclusion criteria, and treatment method ‎was explained to them.‎

## Results

Three participants dropped out from each group, so the final sample of the study was 44, each ‎group consisting 22 participants. The control group included 6 males and 16 females, while the ‎case group included 5 males and 17 female. The mean age of the control group was 35.2 (±9.4) ‎years and it was 30.7 (±7.9) years for the case group. ‎Considering the fact that the participants were selected through convenience sampling, to be able ‎to generalize the study findings, we evaluated the two groups considering the demographic ‎variables including age, sex, and level of education. The results of Chi-square test and ‎independent t-test revealed no significant difference between the two groups in these variables. ‎The mean scores of depression and emotion regulation disorder in the two groups before and ‎after the intervention are presented in the following graphs. ‎

**Figure1:**
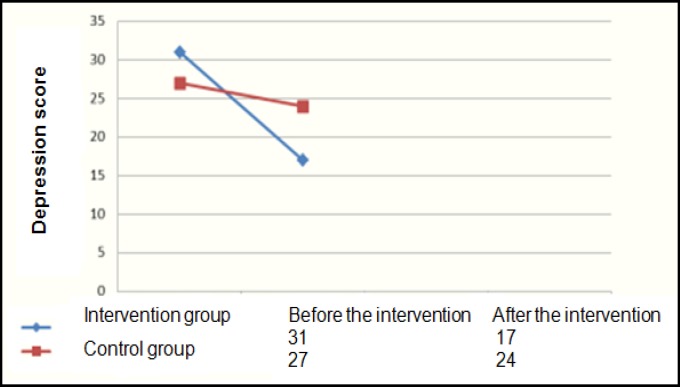
The Mean Changes in Depression Scores Pre and Post Treatment

As the Fig. 1 demonstrates, the intervention group’s depression scores considerably decreased in ‎the post-test compared to the pre-test. The decrease was statistically significant (P = 0.000).‎

**Figure2:**
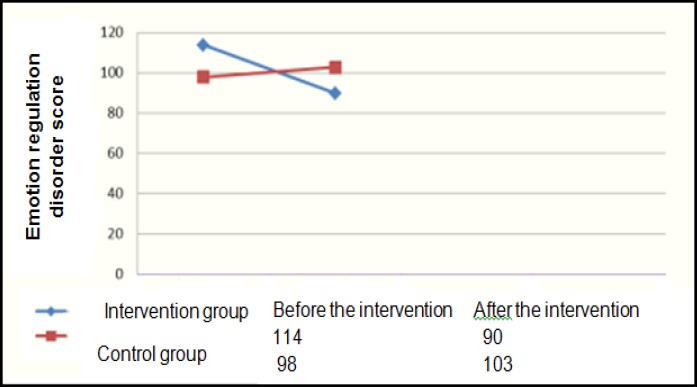
The Mean Changes in Emotion Regulation Disorder Pre and Post Treatment

Fig. 2 displays a considerable decrease in the intervention group’s mean score of emotion ‎regulation disorder in the post-test compared to the pre-test. On the other hand, a slight increase ‎could be observed in the mean score of the control group. The two groups were independently ‎considered in the analyses, so ANCOVA was used to control the effect of the pre-test. Before ‎performing ANCOVA, the variables were investigated concerning the necessary assumptions, ‎and the results revealed that parallelism of the slope of the regression line and normal distribution ‎of the data applied to the data in both groups. ‎

According to the following table, a significant difference was found between the two groups in ‎the emotion regulation disorder scores in the pre-test and the post-test (F = 7.2, P = 0.01). ‎Moreover, the study results revealed a significant difference between the two groups in the ‎subscales of “not accepting the emotional responses”, “problem in impulse control”, and ‎‎“limitation in finding emotion regulation strategies”.

**Table1:** Curriculum of each Intervention Sessions’ content

**Sessions**	**Contents of each session**
Session 1	Establishing orientation of the class and setting the rules, raisin exercise was used to train the participants to concentrate on the present moment; body scan practice; breath focus exercise
Session2	Body scan practice; thought and feeling exercise; pleasant event calendar, mindfulness of routine activities
Session 3	Seeing and hearing exercises; sitting meditation; a three-minute breathing space; mindful walking; unpleasant event calendar
Session 4	Seeing and hearing exercises; sitting meditation; defining the territory of depression: Negative automatic thoughts; diagnosis criteria for depression
Session 5	Sitting meditation; breathing space; reading poems related to mindfulness; introducing the concept of “acceptance”
Session 6	Sitting meditation; mood; thoughts and alternative points exercise; breathing space, observing thoughts and feelings technique
Session 7	Sitting meditation; exercise to explore links between activity and mood; behavioral activation (generate a list of pleasure and mastery activities); identifying actions to take in low mood periods
Session 8	Body scan practice; reviewing the whole course; discussing how to keep up with what was developed over the past seven weeks; discussing plans and positive reasons for maintaining the practice

**Table2:** The Results of ANCOVA on the Groups' Mean Scores of Emotion Regulation Post-Test

**Source of change**		**Sum of squares**	**Degree of freedom**		**Mean squares**		**F coefficient**	**P-value**
**Subscales**	Not accepting the emotional responses	185.2	1		185.2		5.2	0.02^*^
	Problem in performing purposeful behaviors	21.8	1		21.8		1.4	0.24
	Problem in impulse control	120.7	1		120.7		6.6	0.01^*^
	Lack of emotional awareness	63.8	1		63.8		3.6	0.06
	Limitation in finding emotion regulation strategies	147.3	1		147.3		6.8	0.01^*^
	Lack of emotional clarity	2.1	1		2.1		0.15	0.69
Emotion regulation disorder		2557.8		1		2557.8	7.2	0.01

However, no significant difference was ‎observed ‎between the two groups in the subscales of “problem in ‎performing purposeful ‎behaviors”, “lack of emotional ‎awareness”, and “lack of emotional clarity”.‎

## Discussion

Studies have shown that depressed individuals have problems in neglecting the loss-related ‎emotional signs and distracting their attention from them. In other words, they are considerably ‎incapable of regulating their emotions after a loss or failure, which is a critical area in ‎depression (10). As Figure 1 depicts, after the intervention, the mean score of depression in the ‎case group significantly differed from that of the control group. The mean scores of depression ‎decreased from severe to mild based on BDI-II in the case group, while the mean scores of the ‎control group was average based on BDI-II both before and after the intervention.‎

The results of Figure 2 revealed a decreasing trend in the case group in terms of emotion ‎regulation variable after receiving MBCT intervention. This result is consistent with the findings ‎of Erisman, Salters-Pednault, and Roemer (n.d) that found levels of mindfulness have a ‎significant relationship with emotion regulation measured by Difficulties in Emotion Regulation ‎Scale (DERS) (31). Thus, it seems that MBCT provides the individuals with dysthymia with more ‎efficient and effective strategies for emotion regulation.‎

Prior to the emergence of acceptance-based approaches, controlling emotions was a proposed ‎way to manage them; however, studies have shown that suppressing emotions to regulate them ‎results in more negative consequences such as poor social adjustment and well-being (32). ‎Repressed emotions also underlie experiential avoidance which means trying to avoid experience ‎of emotions, feelings, thoughts and other mental contents (33) and studies have shown it to have ‎a critical role in levels of anxiety disorders and human suffering such as prolonging the course of ‎depression (34, 35). Therefore, emotional acceptance by adopting a non-judgmental and ‎welcoming stance toward internal experiences have been encouraged in stark contrast to ‎struggling to eliminate, control or changing emotions (32). This concept has been discussed ‎extensively in the context of mindfulness-based theories. Mindfulness-based therapies by ‎changing one's relationship with his/ her experiences and cultivating the notion of acceptance ‎provides strategies for effective emotion regulation and self-regulation (36). Furthermore, facing with ‎unpleasant emotional states repeatedly, which is broadly encouraged in MBCT, is also a kind of ‎exposure with these unwanted mental contents to the goal of extinction. Apparently, it differs ‎from cognitive therapy approaches in that the focus is on altering the content of thoughts (31, ‎‎^37^).‎

The results of Table 2 showed that among the emotion regulation subscales, the main changes ‎could be attributed to three subscales of “acceptance of positive or negative emotions”, “problem‎ in impulse control” and “individuals’ finding strategies for regulating these emotions”. In line ‎with recent studies that propose emotional experiences rather than suppression as an effective ‎strategy for emotion regulation (32, 34, 37-39), we can state that increasing one’s acceptance ‎toward his/ her emotional responses can broaden one’s mental space to search for new solutions to ‎manage internal impulses and external events. More recent theories of emotion regulation ‎emphasize that identification, acceptance, and being aware of the emotions lead to selecting the ‎most appropriate way for achieving the intended goals. The results also revealed that accepting ‎emotions rather than suppressing and denying them, impede self-blaming in the individual, a ‎component that studies found to create more problems in emotion regulation (11). ‎

On the other hand, the significant decrease in the mean score of “problem in impulse control” in ‎the intervention group showed the difference between experiencing an emotion and acting on its ‎base, which is highly emphasized in mindfulness interventions. For most people, experiencing an ‎emotion means acting upon it or suppressing it. In addition, mindfulness exercises and ‎meditations provide the individual with various choices to replace the usual inefficient strategies ‎when experiencing negative emotion-focused strategies used to distract from the present condition ‎and focusing on other environmental components. This can be confirmed by significant changes ‎in the scores of “problem in impulse control” and “limitation in finding emotion regulation ‎strategies” subscales. ‎

With respect to the other subscales, no statistically significant difference was observed between ‎the two groups in “lack of emotional clarity” subscales in the post-test. These subscales evaluate ‎the distinction among the emotions as well as the emotions’ verbal description, which seems to be ‎less affected by the mindfulness training. It seems that the change in the regulation of emotions ‎was affected by factors other than identifying accurate emotions. In the Iranian culture, depressed ‎people tend to report and recognize their mental contents as thoughts rather than feelings. Patients’ ‎inability to differentiate thoughts from emotions might be due to the inseparable nature of ‎differentiating thoughts, emotions or other mental contents as mindfulness literature did not make ‎such a distinction either (31). Wilber (1996) proposes that "information processing occurs ‎hierarchically across cognitive, emotional and physiological systems, which have been suggested ‎to be functionally interdependent and inseparable" (31). However, more researches are needed to ‎enable us to interpret this result accurately.‎

As no significant change was found in the “problem in performing purposeful behaviors” subscale, ‎and because our sample was a group of dysthymic patients, we dealt with two delineating factors of ‎chronic course of depression and chronic use of antidepressants, both of which are accompanied ‎by apathy features. Hence, transferring cognitive and emotional changes into behavioral ones can ‎be attributed to these features. The short-term course of the treatment can also be another reason ‎because converting the adaptive alternatives for emotional regulation into practical solutions to the ‎extent that the individuals can overcome their daily hassles is a time consuming process and needs ‎more long-term interventions. Moreover, making use of cognitive-behavioral interventions such as ‎self-control and positive reinforcement programs together with mindfulness might be ‎accompanied by more comprehensive effectiveness.‎

## Limitations

Non-random selection of this study and lack of follow-up due to time constraints were two of ‎the study limitations. Therefore, conducting more studies on this topic is highly recommended to ‎generalize the findings and evaluate the maintenance of the results over time. Moreover, the type ‎and dosage of antidepressant medication were not controlled in the two groups. Therefore, it ‎seems that evaluating the efficacy of MBCT combined with specific dosage or group of ‎antidepressants could more accurately determine the optimal mode of the combination therapy. ‎Furthermore, we suggest that anxiety, which seems to be a moderating factor in the effectiveness ‎of treatment be controlled in the future studies.‎

## Conclusion

In this study, it was found that the severity of depression and the ability of emotion regulation of ‎patients with dysthymia who had participated in MBCT sessions improved significantly ‎compared to the patients who did not participate in the program. Hence, it may be argued that ‎mindfulness skills could show their effects in relation to chronic depression, in which emotion ‎regulation has a central role.‎
